# Beriberi Neuropathy Mimicking Guillian-Barre Syndrome in a Teenager With Food Restriction: A Case Report

**DOI:** 10.7759/cureus.21417

**Published:** 2022-01-19

**Authors:** Hannah Morrissey, Farman Ali, Collin John, Gauri Pawar, Elizabeth A McQuade

**Affiliations:** 1 General Pediatrics, West Virginia University Medicine, Morgantown, USA; 2 Adult Neurology, West Virginia University Medicine, Morgantown, USA; 3 Internal Medicine/Pediatrics, West Virginia University Medicine, Morgantown, USA; 4 Child Neurology and Neurodevelopmental Disabilities, West Virginia University Medicine, Morgantown, USA

**Keywords:** progressive weakness, vitamin b1 deficiency, nutritional neuropathy, wernicke encephalopathy, eating disorder, guillian barre syndrome, gbs mimics, thiamine neuropathy, dry beriberi, thiamine deficiency

## Abstract

Beriberi neuropathy (thiamine deficiency) and Guillian-Barre Syndrome (GBS) both can present with areflexia and progressive ascending weakness. A physical examination can be equivocal between the two. In cases where GBS is suspected clinically but initial work-up with cerebral spinal fluid (CSF) studies and magnetic resonance imaging (MRI) of the spine are not diagnostic, nerve conduction study/electromyography (NCS/EMG) should be done to evaluate beriberi neuropathy. Presumptive treatment should be started while awaiting confirmation from nutritional laboratory investigations. Here we present a rare case of a GBS mimic involving a 17-year-old patient with food restriction that led to thiamine deficiency causing beriberi neuropathy and Wernicke encephalopathy.

## Introduction

Thiamine (vitamin B1) is a nutrient that must be consumed by adequate dietary intake through both meat and plant-sourced foods [1-3]. A patient with dysfunctional eating can have a normal weight and body mass index (BMI), thus a dietary history can aid diagnostic suspicion. Thiamine deficiency is known as beriberi, with subtypes of wet (with cardiac failure) and dry (without cardiac failure) [2]. Beriberi can present with neurologic complications including peripheral neuropathy, hoarseness, and Wernicke encephalopathy which were all seen in our case [1]. 

## Case presentation

A 17-year-old female with obesity presented after one week of progressive ascending weakness and paresthesias in her extremities. Two weeks prior, she had nausea, vomiting, abdominal pain, diagnosed as biliary colic. One week prior she developed tingling in her feet that spread to her ankles. Over the next few days, this turned to weakness in her feet which then spread to proximal leg muscles causing unsteady gait and frequent falls. Two days prior to presentation she developed progressive leg weakness so that she was unable to walk at the time of presentation, and developed new paresthesias in her fingers. She denied bowel/bladder incontinence but complained of dysphagia. She described both, purging after meals and restrictive eating over the last six months due to her concerns about body image, for which she has never sought medical treatment. She lost 66 lbs over the last one month. 

On initial examination, she weighed 173 lbs (body mass index {BMI} 27.10). Blood pressure was 141/96 mmHg, heart rate was 115 beats per minute, respiratory rate 15 breaths per minute, temperature 36.5 degrees celsius, and 100% oxygen saturation. On cardiac examination, she was tachycardic but was without murmur. Pulmonary and gastrointestinal examinations were normal. Neurological examination revealed that she was alert and responsive; however, she had a child-like effect with hypophonia. Her cranial nerves were intact, without nystagmus. Strength in upper extremities was 4/5 proximally and 2/5 distally, and 3/5 proximally and 1/5 distally in the lower extremities. Examination revealed length-dependent reduced fine touch and pinprick below both ankles and both wrists bilaterally. Joint position sense was not intact in all extremities. Deep tendon reflexes were 1+ in the upper extremities, absent at the knee and ankle, with equivocal plantar reflexes. She was unable to ambulate. 

The basic metabolic panel, complete blood count, urinalysis, urine drug screen, serum human chorionic gonadotropin, lipase, and erythrocyte sedimentation rate were within normal limits. Creatinine kinase was elevated at 971 (reference {Ref} range: 30-135 U/L), aspartate transaminase (AST) was elevated at 53 (Ref range: 8-41 U/L), alanine transaminase (ALT) elevated at 87 (Ref: <55 U/L). Magnetic resonance imaging (MRI) of the brain and spine with and without contrast showed no stroke or white matter plaques. A lumbar puncture revealed cerebrospinal fluid (CSF) with 1 nucleated cell/uL and 0 red blood cell/uL, protein 20 mg/dL, and glucose 59 mg/dL. 

She was given a presumptive diagnosis of early-onset Guillian-Barre Syndrome (GBS) with bulbar involvement and was treated with intravenous immunoglobulin (IVIG). The patient worsened following treatment with two doses of IVIG. The patient’s therapy was changed to plasma exchange therapy (PLEX). Despite this change of treatment, she developed new-onset bilateral horizontal nystagmus, which raised suspicion for a central process in addition to her peripheral neuropathy. She remained alert and did not appear confused when asked simple questions; however, she did have a persistent "child-like" effect that was incongruent with her age. A nerve conduction study (NCS) with electromyography (EMG) revealed low amplitudes in the motor nerve action potentials in upper and lower extremities consistent with axonal neuropathy. Although GBS rarely has an axonal variant, the most common pattern on NCS is demyelination which was not found. In addition, needle EMG showed signs of active denervation in lower extremity muscles indicating a chronic process, whereas GBS would be an acute process [4,5]. These findings, in addition to the previous nondiagnostic CSF and MRI spine, ruled out GBS. It was thus suspected that her symptoms were due to chronic axonal neuropathy. 

**Figure 1:**
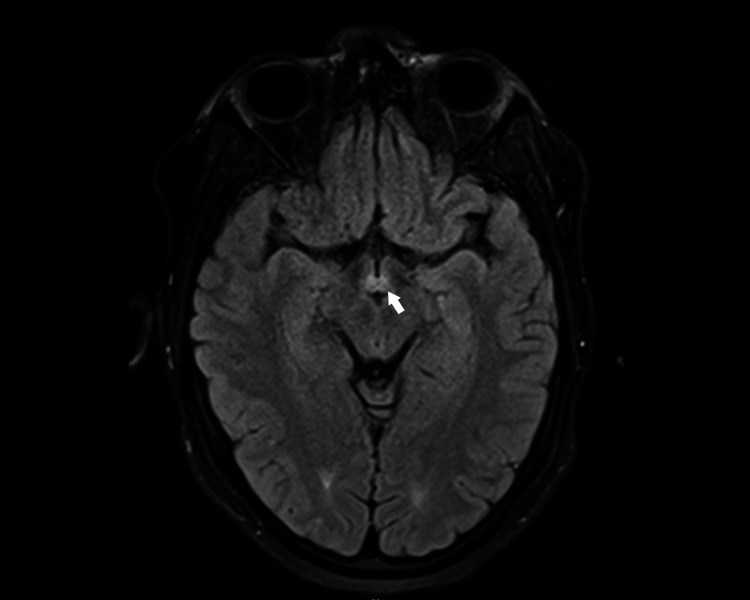
MRI brain White arrow: axial T2 flair showing T2 hyperintensity of the mammillary bodies bilaterally

The MRI of the brain was reassessed and subtle increased signal intensity of mamillary bodies was noted (Figure 1). Vitamin B12, folate, methylmalonic acid, homocysteine, vitamin E, copper, and zinc levels were obtained and were found normal. Thiamine levels were significantly below normal at a level of 24 (Ref range: 70-180 nmol/L) revealing her diagnosis of dietary thiamine (vitamin B1) deficiency secondary to her eating disorder, with Wernicke encephalopathy and beriberi. She had a normal echocardiogram and normal thyroid-stimulating hormone (TSH) levels. It was suspected that her tachycardia, hypertension, and widened pulse pressures were the beginning of high output cardiac failure from progression from dry to wet beriberi. 

The patient was treated with intravascular thiamine 500 mg for three days, then oral thiamine 250 mg daily for five days, and then 100 mg daily until reevaluated in follow-up. Because symptoms of vitamin deficiency can be present even with normal levels, she was treated with daily multivitamin, vitamin E, and B complex vitamins (comprised of thiamine, riboflavin, niacin, niacinamide, pyridoxine, biotin, pantothenic acid, folic acid, and vitamin B12) [1]. Gabapentin was used to treat her neuropathic symptoms. She started on antihypertensive medication. 

Her symptoms stabilized after thiamine therapy. At discharge, her neuropathic pain, dysphagia, and hypophonia completely resolved. Her nystagmus was still present, although subtle. She had +1 reflexes in the upper extremities; however, remained areflexic in the lower extremities. Her numbness and weakness had improved significantly, but she was still using a walker to ambulate. A psychiatric follow-up was established for her eating disorder and she was discharged to an inpatient rehabilitation facility. 

Clinic reassessment occurred five months after discharge. Her neurologic examination at that time showed no nystagmus, normal reflexes throughout, normal position sense. She continued to report faint numbness at her fingertips. Strength in upper extremities was 5/5 proximally and 4/5 distally and 5/5 proximally and 4/5 distally in the lower extremities. She ambulated without the use of an assistive device. Thiamine levels were within normal limits at 154 (Ref range: 70-180 nmol/L). 

## Discussion

Our patient’s presentation of gastrointestinal symptoms two weeks prior, followed by progressive weakness, sensory changes, lower extremity areflexia, dysphagia, and voice changes made GBS likely. The typical spinal fluid shows albumino-cytologic dissociation (increased total protein with normal total nucleated cell count) in GBS, however, it can be normal in 30-50% of the cases early in presentation and 10-30% of patients in the second week of symptoms [4]. Thus the patient's normal CSF analysis did not rule out a diagnosis of GBS. Enhancement of the conus medullaris or spinal nerve roots of the cauda equina was not seen on lumbar MRI; however, this too can be seen in patients with GBS [4,5]. Neuromuscular disorders such as myasthenia gravis or botulism can also present with acute and progressive weakness; however, the prominent sensory symptoms in our patient also made this unlikely. Functional neurological disorders can present as a combination of physical, sensorimotor, and cognitive symptoms. Our patient did have an abnormal effect with an initial unremarkable work-up; however, her physical examination was concerning for an organic process. Functional disorders can co-occur with organic diseases; however, the patient’s clinical picture was not consistent with a functional neurologic disorder. Ultimately, our patient was confirmed to have thiamine (vitamin B1) deficiency secondary to her eating disorder, with Wernicke encephalopathy and beriberi. 

Vitamin B1 is a water-soluble micronutrient found in both meat and plant-sourced foods [1,2]. There is no endogenous synthesis of thiamine in the human body, and if intake is inadequate, one can become thiamine deficient within two to three weeks [1,3]. Inadequate dietary intake is the most common cause [2,6], and patients with alcohol abuse, eating disorders, total parental nutrition, older age, pregnancy, homelessness are at increased risk [1,2]. Other less common causes of deficiency include poor intestinal absorption or increased losses, including conditions such as short gut syndrome, post-bariatric surgeries, severe burns, septic shock, and refeeding syndrome [2,3]. 

Thiamine deficiency is known as beriberi, which can be classified as either wet (with cardiac failure) or dry (without cardiac failure) [2]. Dry beriberi presents as a peripheral neuropathy characterized by areflexia and both sensory and motor impairments primarily involving distal extremities, starting in the legs and ascending to hands, which can mimic GBS [1]. It may also cause hoarseness due to the involvement of the recurrent laryngeal nerve as was seen in our patient [1]. Nerve conduction studies demonstrate axonal generalized peripheral neuropathy without conduction blocks, temporal dispersion, and no prolongation of distal motor or F-wave latencies typically seen in GBS [4,5]. 

Wernicke's encephalopathy may be coexistent with about 25% of cases of beriberi [1]. Wernicke’s encephalopathy is characterized by a triad of nystagmus/ophthalmoplegia, ataxia, and confusion; however, it is not necessary for all symptoms to be present [2,6]. Confabulation, apathy, and amnesia can then develop into the chronic form of Korsakoff syndrome [6]. Typical findings of the MRI brain include increase signal alterations in the thalami, mammillary bodies, tectal plate, and periaqueductal area [3,6]. Cortical lesions are rare and concerning for poorer outcomes [6]. 

The prognosis for beriberi is reassuring, with anticipated recovery taking three to six months. Often, motor symptoms recover more fully than sensory symptoms, however, if severe there can be permanent deficits [1,6]. A significant, if not complete recovery from Wernicke’s encephalopathy is anticipated with adequate treatment, and there have been no pediatric reports of Korsakoff syndrome. However, Wernicke’s encephalopathy/Korsakoff syndrome is an under-diagnosed entity and the full impact of its effects may not be known in children [6]. 

## Conclusions

Both GBS and beriberi neuropathy can present with areflexia and progressive ascending weakness. If CSF studies and MRI spine are not diagnostic, NCS/EMG should be done. Eating habits, alcohol abuse, homelessness status, or underlying conditions for gastrointestinal losses are important diagnostic clues for thiamine deficiency. Nutritional deficiencies and eating disorders can present in normal-weight individuals. Patients can present with altered mental status or personality changes as early signs of encephalopathy, however, these presentations can be subtle. Presumptive treatment for nutritional deficiencies has a high benefit over risk profile and treatment should be initiated before investigative results are obtained to prevent worsening of disease that may be irreversible in some patients. 
